# HYFI: Hybrid filling of the dead‐time gap for faster zero echo time imaging

**DOI:** 10.1002/nbm.4493

**Published:** 2021-02-23

**Authors:** Romain Froidevaux, Markus Weiger, Manuela B. Rösler, David O. Brunner, Klaas P. Pruessmann

**Affiliations:** ^1^ Institute for Biomedical Engineering ETH Zurich and University of Zurich Zurich Switzerland

**Keywords:** bone, gap filling, PETRA, short *T*_2_, SNR efficiency, SPI, WASPI, ZTE

## Abstract

The aim of this work was to improve the SNR efficiency of zero echo time (ZTE) MRI pulse sequences for faster imaging of short‐*T*_2_ components at large dead‐time gaps. ZTE MRI with hybrid filling (HYFI) is a strategy for retrieving inner k‐space data missed during the dead‐time gaps arising from radio‐frequency excitation and switching in ZTE imaging. It performs hybrid filling of the inner k‐space with a small single‐point‐imaging core surrounded by a stack of shells acquired on radial readouts in an onion‐like fashion. The exposition of this concept is followed by translation into guidelines for parameter choice and implementation details. The imaging properties and performance of HYFI are studied in simulations as well as phantom, in vitro and in vivo imaging, with an emphasis on comparison with the pointwise encoding time reduction with radial acquisition (PETRA) technique. Simulations predict higher SNR efficiency for HYFI compared with PETRA at preserved image quality, with the advantage increasing with the size of the k‐space gap. These results are confirmed by imaging experiments with gap sizes of 25 to 50 Nyquist dwells, in which scan times for similar image quality could be reduced by 25% to 60%. The HYFI technique provides both high SNR efficiency and image quality, thus outperforming previously known ZTE‐based pulse sequences, in particular for large k‐space gaps. Promising applications include direct imaging of ultrashort‐*T*_2_ components, such as the myelin bilayer or collagen, *T*_2_ mapping of ultrafast relaxing signals, and ZTE imaging with reduced chemical shift artifacts.

## Graphical abstract

HYFI is a strategy for retrieving inner k‐space data missed during the dead‐time gap arising from radio‐frequency excitation and switching in ZTE imaging. It performs hybrid filling of the inner k‐space with a small single‐point‐imaging core surrounded by a stack of shells and provides both high SNR efficiency and image quality, thus outperforming previously known ZTE‐based pulse sequences, particularly at large dead‐time gaps. Promising applications include direct imaging of ultrashort components, such as the myelin bilayer or collagen.

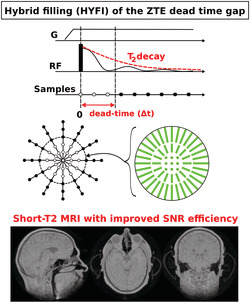

**Abbreviations used**
FID: free induction decay
FOV: field of view
HYFI: ZTE MRI with hybrid filling
MTF: modulation transfer function
PETRA: pointwise encoding time reduction with radial acquisition
PSF: point spread function
RHE: ramped hybrid encoding
SNR: signal‐to‐noise ratio
SPI: single‐point imaging
SWIFT: sweep imaging with Fourier transformation
ZTE: zero echo time

## 1 INTRODUCTION

**FIGURE 1:**
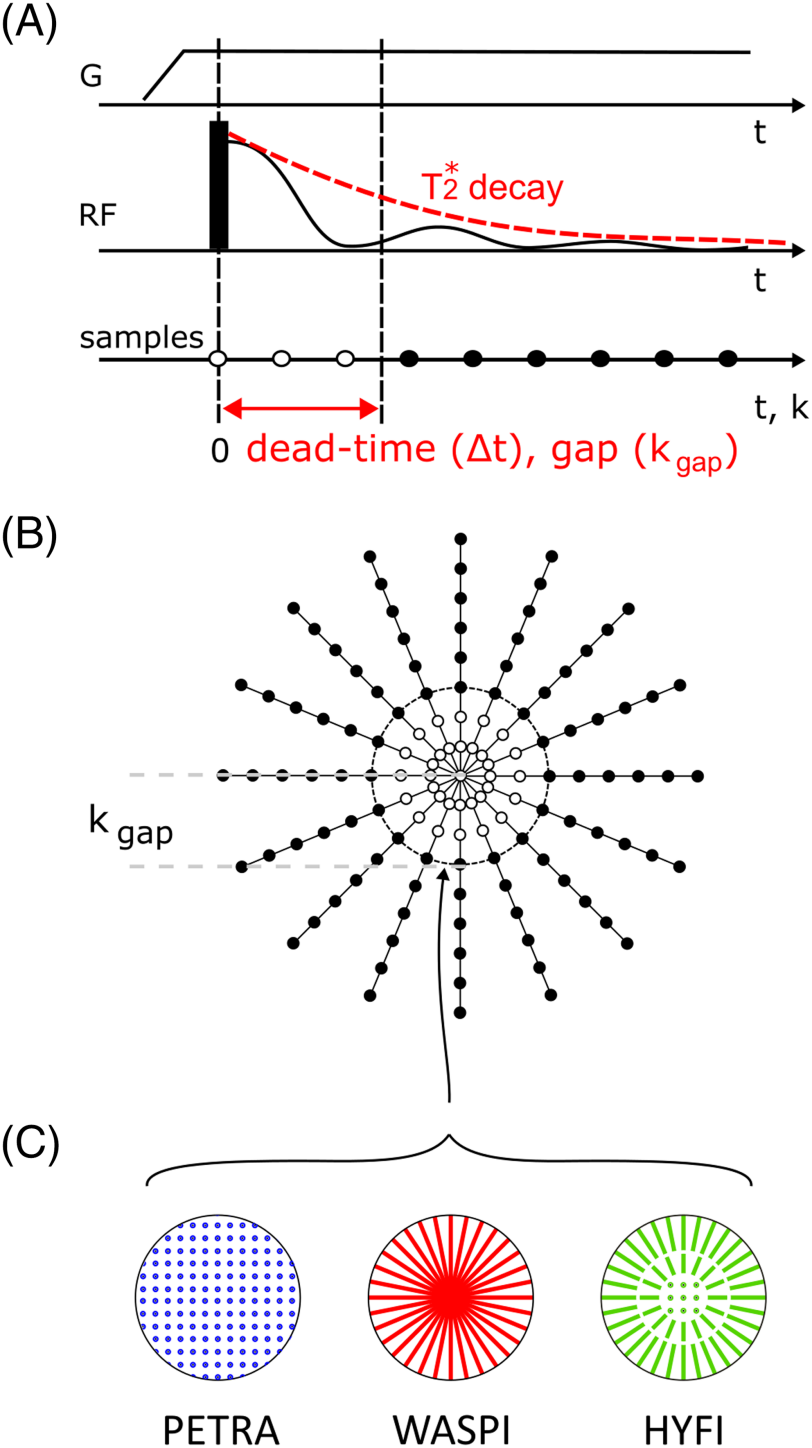
Zero echo time (ZTE) data acquisition. (A) The gradient G applied for radial center‐out encoding is ramped up before radio‐frequency (RF) excitation. The initial part of the signal cannot be acquired due to the dead time Δt (white dots), which leads to a k‐space gap of size k_gap_. (B) After each excitation, a different radial spoke is acquired to fill a 3D k‐space volume. The dead time leads to a spherical gap of radius k_gap_ in the center of k‐space. (C) PETRA, WASPI and HYFI all provide the missing data using additional acquisitions at lower gradient strengths. However, these techniques differ in acquisition timing and geometry. In PETRA, the inner k‐space (inside the gap) is acquired single‐pointwise on a Cartesian grid. In WASPI, a second set of radial acquisitions is performed with strongly reduced gradient strength. HYFI consists of a Cartesian single‐point core surrounded by several radially acquired shells. Note that the figure partially reuses schemes of previous publications by the same author^22^, ^23^

Direct MRI of tissues with very short transverse relaxation times *T*_2_ or *T*_2_** in the submillisecond range, such as bone,^1^, ^2^, ^3^ tendon,^4^, ^5^, ^6^ myelin,^7^, ^8^, ^9^, ^10^ lung^11^, ^12^, ^13^ and teeth,^14^, ^15^, ^16^ is receiving increasing attention due to its potential for both clinical diagnosis and basic research. The rapid signal decay of such tissues prevents detection and spatial encoding through conventional echo‐based sequences. Therefore, several dedicated short‐*T*_2_ techniques have been developed, usually avoiding echo formation.^17^ One efficient and increasingly used technique is zero echo time (ZTE) imaging,^18^, ^19^, ^20^, ^21^ where a frequency‐encoding gradient is switched on before radio‐frequency (RF) excitation and signal is acquired as soon as possible afterwards (Figure 1A). In this way, 3D k‐space is covered with radial center‐out trajectories and spherical support (Figure 1B).

In ZTE sequences, the dead time Δ*t* separating signal excitation and reception prevents acquisition of early data and has a lower limit determined by half the RF pulse duration, transmit‐receive switching and the filter group delay. This leads to a gap in central k‐space,^20^ (i.e. no data are available in a sphere of radius *k*_*gap*_ centered on the k‐space origin). To avoid related image artifacts, three approaches have been suggested: (a) generating the missing information through algebraic reconstruction^24^; (b) recovering it through additional acquisitions using Cartesian single‐point imaging (SPI),^25^, ^26^ as in the pointwise encoding time reduction with radial acquisition (PETRA) technique^21^; and (c) recovering the data by radial readouts at lower gradient strength, as in the WASPI technique (water‐ and fat‐suppressed proton projection MRI)^19^ (Figure 1C).

Algebraic ZTE has the advantages of not requiring additional acquisition and having a benign behavior of the point spread function (PSF) under *T*_2_* decay. However, image reconstruction becomes ill‐conditioned for *k*_*gap*_ exceeding three Nyquist dwells (dk) (where dk = 1/FOV and FOV is the field of view),^27^ hence preventing application at larger gaps. WASPI retrieves the missing data with a time‐efficient radial acquisition, which, however, leads to a discontinuous *T*_2_ *‐related modulation transfer function (MTF) in k‐space and thus a propensity for increased PSF side lobes and associated oscillatory image artefacts.^22^ On the other hand, PETRA is robust against artifacts but hampered by the slow SPI acquisition of the inner k‐space (k < *k*_*gap*_).^22^ Therefore, the best sequence choice depends on the particular imaging task and especially on the gap size. However, none of the described methods are well suited for large gaps (i.e. *k*_*gap*_ of tens of dwells), because algebraic ZTE and WASPI lead to poor image quality and PETRA causes undesirably long scan times. Yet, imaging under such conditions can be necessary or beneficial. Indeed, large gaps occur at high imaging bandwidth, as required for high‐resolution imaging of short‐*T*_2_ components, in particular in large FOVs,^23^ or when the dead time is relatively large, either because of limitations of the RF hardware or by choice to enable *T*_2_ * selection,^21^ *T*_2_* mapping, or reduction of chemical‐shift artefacts.

In this work, we explore hybrid filling (HYFI) of the k‐space center as a path to ZTE imaging that is both time‐efficient and robust despite large k‐space gaps. The hybrid approach, which reconciles the advantages of point‐wise and radial filling, was recently sketched in a conference presentation.^28^ In the meantime, it has shown promise in a first applied study.^10^, ^29^ On this basis, the present work has the dual aim of establishing the technical basis of HYFI in due detail and assessing its imaging properties and SNR efficiency in comparison with PETRA. This is done by PSF analysis, 3D imaging simulations, and phantom as well as in vivo imaging.

## 2 METHODS

### 2.1 Hybrid filling

The basic idea of HYFI is to replace the SPI part in PETRA by a more time‐efficient acquisition strategy with a pattern that avoids strong discontinuities in the MTF, as occurring in WASPI. This is accomplished by using radial acquisitions, but with a short acquisition time to restrain *T*_2_* decay. To fill the dead‐time gap under this condition, multiple sets of radial acquisitions are used with decreasing gradient strengths. However, around the origin, the encoding speed is so small that the distance traveled within an adequate time is below the distance separating two data points,^†^ dk. In such circumstances, data are acquired single pointwise. This approach results in a hybrid filling of the inner k‐space with a small spherical SPI core surrounded by a stack of shells acquired on radial readouts in an onion‐like fashion (Figure 1C).

**FIGURE 2:**
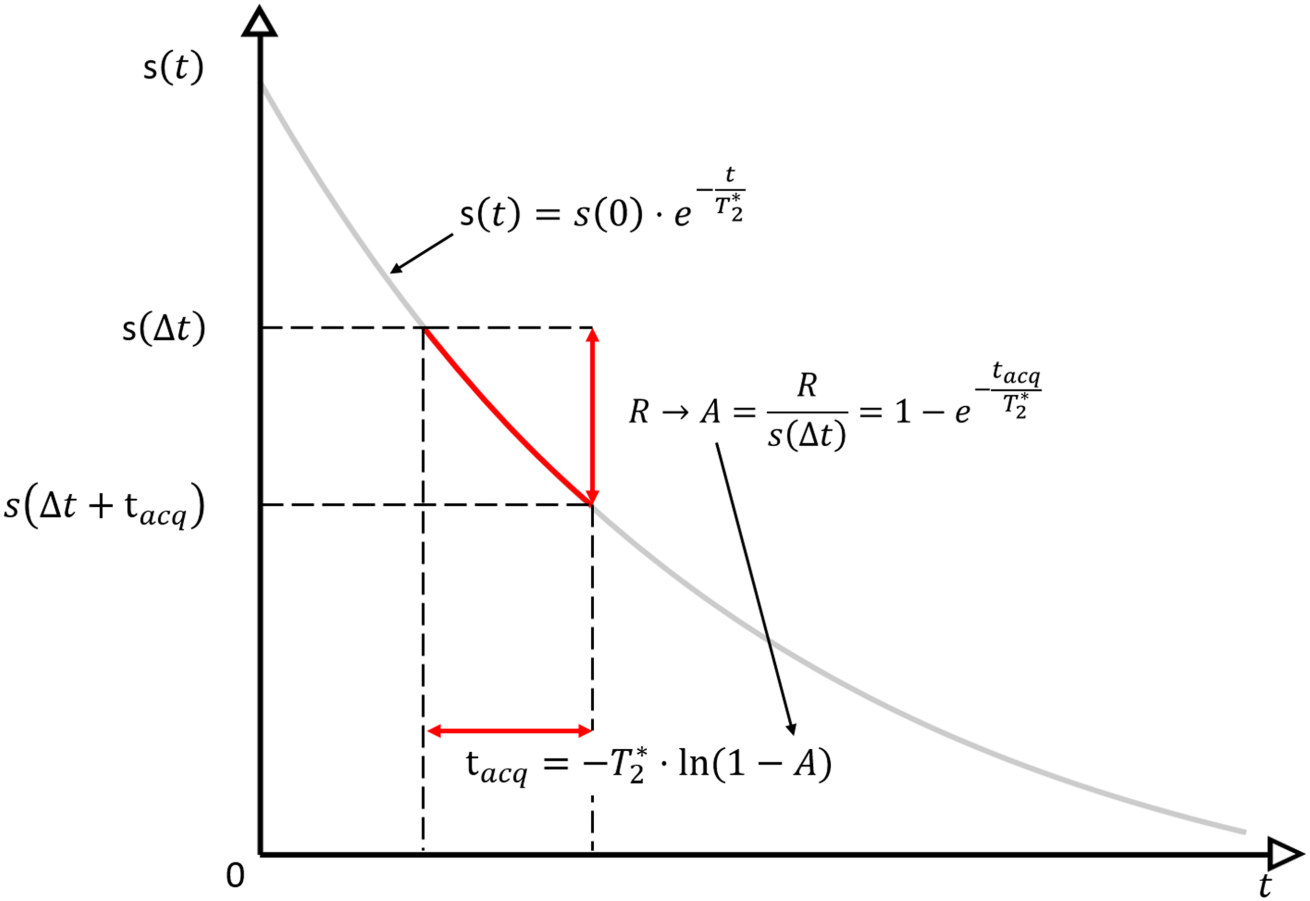
Basic principle of HYFI acquisition to avoid strong discontinuities in the recorded signal amplitudes. Due to *T*_2_* relaxation, the MR signal *s* decays exponentially over time. With the zero echo time (ZTE) sequence of Figure 1A, acquisition starts after the dead time Δ*t*. In HYFI, any subsequent data collection for the inner k‐space is restricted to an acquisition time *t*_*acq*_ in order to limit signal amplitudes to a given range *R*. Normalization of *R* by the signal amplitude at the dead time *s*(Δ*t*) provides the amplitude coefficient *A*. In practice, a target *T*_2_* and *A* are selected and *t*_*acq*_ follows from the given equation

To develop an algorithm implementing the principle outlined above, a range *R* is introduced, to which amplitudes of decaying signals are limited (Figure 2). Dividing *R* by the signal amplitude at the dead time Δ*t* leads to the amplitude coefficient *A* describing the allowed range of signal amplitudes in a normalized way. For a given signal decay, selecting a value for *A* results in *t*_*acq*_, the maximum duration an acquisition may last after Δ*t*. Figure 2 shows that for exponential signal decay as assumed throughout this work follows

(1) $$t_{\mathrm{acq}}=-T_{2}^{*}\;\ln\left(1-A\right).$$

Based on this parameter, the pattern and timing of the inner k‐space acquisition are derived (i.e. the number and boundaries of concentric subvolumes as well as associated gradient strengths). The gradient strength required to reach a certain k‐value after Δ*t* is

(2) $$G=\frac{k}{\gamma ∆t},$$

whereγ is the gyromagnetic ratio in frequency units. Close to the k‐space origin, small values of *G* are required for the first points on the grid with spacing *dk*. With such slow encoding, only a single data point may then fit into *t*_*acq*_. This situation results in a spherical core region where data are acquired single pointwise, and which are acquired efficiently on a Cartesian grid. The radius *k*_*SPI*_ of the SPI core is determined by the onset of the radial acquisition when the condition *γ G t*_*acq*_ = *dk* is fulfilled, leading by combination with Equation 2 to

(3) $$k_{\mathrm{SPI}}=\frac{\Delta t}{t_{\mathrm{acq}}}\mathrm{dk}.$$

At k‐space radii larger than *k*_*SPI*_, at least two points are acquired during *t*_*acq*_. The associated k‐space volumes are concentric shells of thickness *γ G t*_*acq*_, where *G* is obtained from Equation 2 by setting *k* to the inner shell radius, starting from *k*_*SPI*_. Note that theoretically *k*_*SPI*_ may reach any value between 0 (*A* = 1) and ∞ (*A* → 0), but is in practice rounded to multiples of *dk* and limited to *k*_*gap*_, because it only concerns data located in the inner k‐space.

**FIGURE 3:**
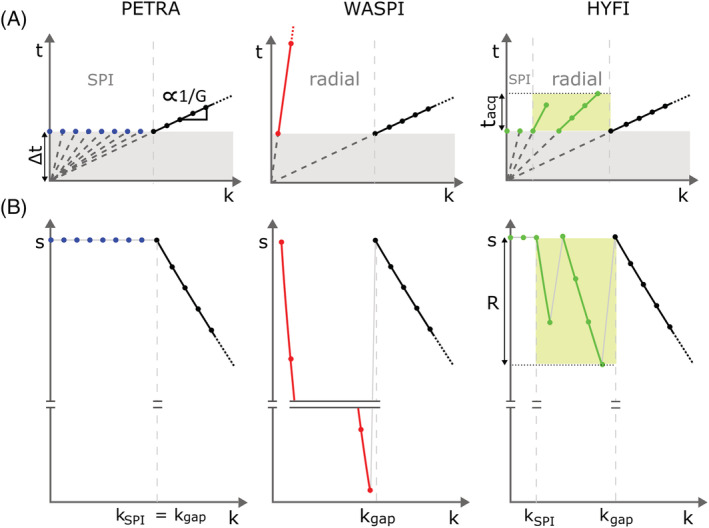
Acquisition timing and *T*_2_* weighting for zero echo time (ZTE)‐based sequences PETRA, WASPI and HYFI. (A) Time of acquisition in k‐space (i.e. the time after excitation when each k‐space point is acquired). For clarity, only part of the positive k‐space is displayed. It includes the inner k‐space (k < k_gap_), colored in blue, red and green, respectively (c.f. Figure 1C), as well as part of the radially acquired outer k‐space in black (c.f. Figure 1B). (B) Modulation transfer functions (MTFs) in k‐space associated with *T*_2_* relaxation. In PETRA, the inner k‐space is acquired with single‐point imaging (SPI) (i.e. every single data point is acquired with a new radio‐frequency excitation and after the same dead time Δ*t*). Different k‐space locations are reached by adapting the gradient strength, which is inversely proportional to the slope of the lines through the origin. In this way, all points in the inner k‐space have the same *T*_2_* weighting, forming a plateau in the MTF. The radially acquired signal in outer k‐space (black line) decays exponentially and is equivalent for all techniques (c.f. Figure 1B). In WASPI, the inner k‐space is acquired radially with strongly reduced gradient strength, creating heavy *T*_2_* weighting, a disruption at k_gap_, and thus image artifacts. In HYFI, all points of the inner k‐space are acquired within a restricted time range *t*_*acq*_ to largely take advantage of the efficiency of radial encoding while limiting signal amplitudes to a range *R* sufficiently small to avoid image artifacts. Only a few central points are acquired with SPI

The HYFI pattern resulting from this procedure is illustrated in Figure 3 in comparison with PETRA and WASPI. Figure 3A shows that in HYFI the inner k‐space is split into subvolumes—the SPI core and a number of shells—such that each of them can be acquired starting after Δ*t* and within *t*_*acq*_. In this way, the amplitudes of exponentially decaying signal are bound to the range *R*, as shown in the MTF in Figure 3B.

To enable deriving *t*_*acq*_, two parameters need to be selected by the operator: (a) the target *T*_2_* represents the approximate *T*_2_* of the tissues of interest and may be chosen using a rule of thumb that is developed in section 2.2; and (b) the amplitude coefficient *A* is set to maximally reduce scan time while keeping artifacts to a negligible level. The optimal value depends on the experimental conditions and can be evaluated by means of PSF calculations (as shown below) or by image simulations (as demonstrated in the Results section). By setting *A* = 0 or *A* = 1, the HYFI algorithm leads to PETRA and WASPI as limiting cases.

**FIGURE 4:**
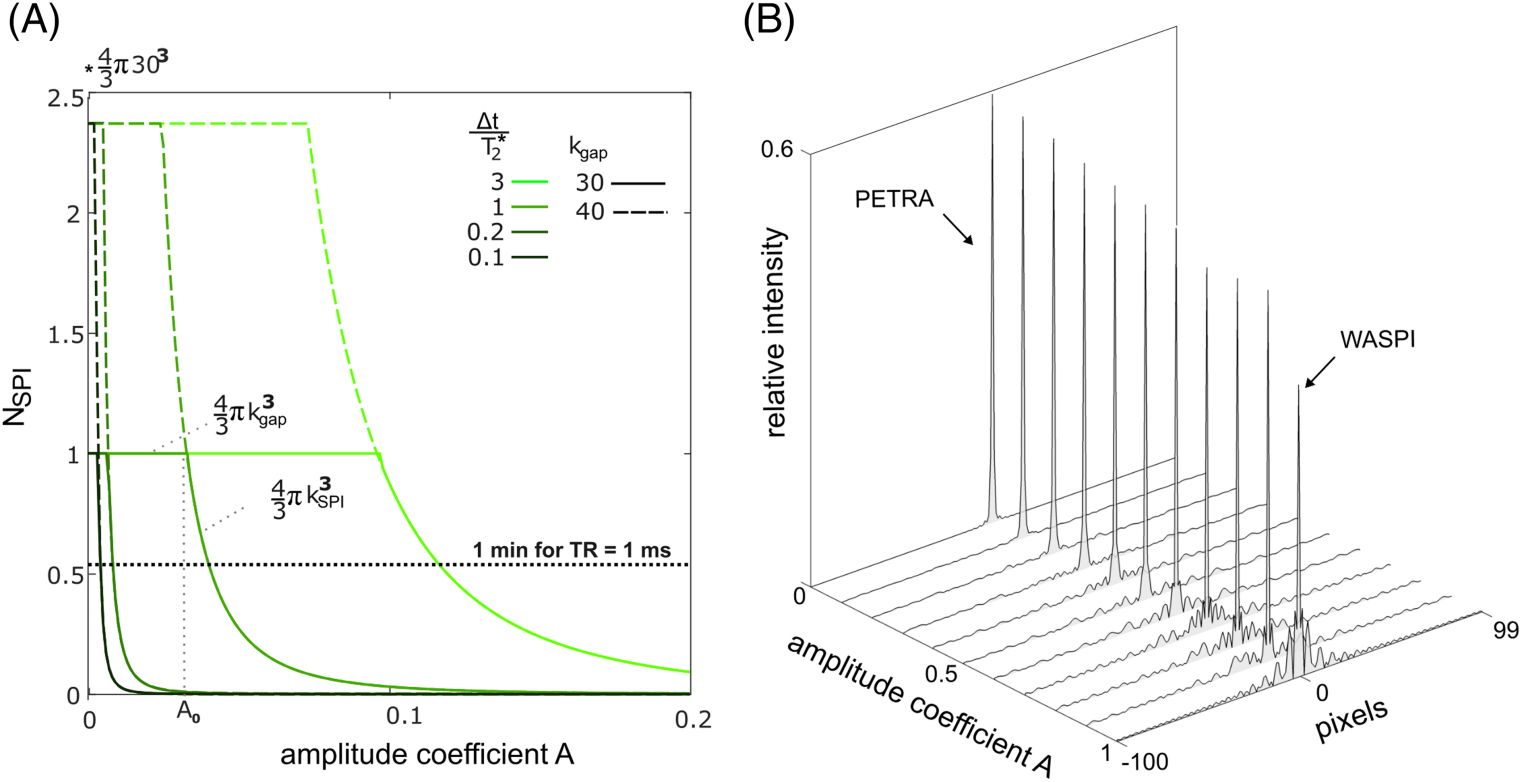
Influence of the HYFI amplitude coefficient *A* on scan time and depiction fidelity. (A) The number of excitations *N*_*SPI*_ required to fill the single‐point imaging (SPI) core largely describes possible savings in scan time. It is plotted as a function of *A* for different ratios Δ*t*/ *T*_2_* and gap sizes *k*_*gap*_. For a given gap and ratio, as *A* increases, *N*_*SPI*_ stays constant up to a particular value $A_{0}=1-\exp\left(-\frac{\Delta t}{T_{2}^{*}\cdot k_{\mathrm{gap}}}\right)$ and then starts diminishing. For *A* < *A*_0_, *k*_*SPI*_ > *k*_*gap*_ and the whole inner k‐space is acquired with SPI, thus requiring $N_{\mathrm{SPI}}=\frac{4}{3}\pi \;k_{\mathrm{gap}}^{3}$ number of radio‐frequency excitations for Cartesian Nyquist sampling.^‡^ For *A* > *A*_0_, part of the inner k‐space can be acquired radially because *k*_*SPI*_ < *k*_*gap*_ and less excitations are needed in the core ( $N_{\mathrm{SPI}}=\frac{4}{3}\pi \;k_{\mathrm{SPI}}^{3}$). In summary, this figure shows that for common situations where Δ*t* is smaller to or comparable with *T*_2_*, the volume of data that needs to be acquired in a time‐consuming SPI manner rapidly decreases to almost negligible values, even with relatively small amplitude coefficient. Assuming a TR of 1 ms, this allows reducing scan time by several minutes compared with PETRA. (B) Point spread function (PSF) lineshapes changing with *A*. Calculations were performed assuming *T*_2_* = 100 dk and *k*_*gap*_ *=* 30 dk. As *A* increases from 0 (PETRA) to 1 (WASPI), the main lobe amplitude decreases and side lobes build up. Importantly, the PSF lineshapes are well preserved for *A* ≈ 0.1, at which *N*_*SPI*_ is substantially decreased. Hence, HYFI is expected to reduce scan time while preserving image quality. More detailed PSFs are shown in Figure S1. Note that PETRA and WASPI acquisitions are also obtained with values of *A* slightly larger than 0 and smaller than 1, respectively (c.f. Matlab script for more details)

Figure 4 illustrates the tradeoff between scan time reduction and PSF quality in the selection of *A*. As savings in scan time are largely described by the reduction of the SPI core, Figure 4A shows the number of excitations *N*_*SPI*_ required to fill the core as a function of *A*. In typical cases where Δ*t* is less than *T*_2_*, choosing *A* ≲ 0.1 is sufficient to decrease the size of the SPI core considerably. Moreover, Figure 4B shows that in such circumstances the PSF lineshape is largely unaffected, suggesting that scan time can indeed be reduced while preserving image quality. More detailed PSFs are shown in the supporting information (Figure S1).

**FIGURE 5:**
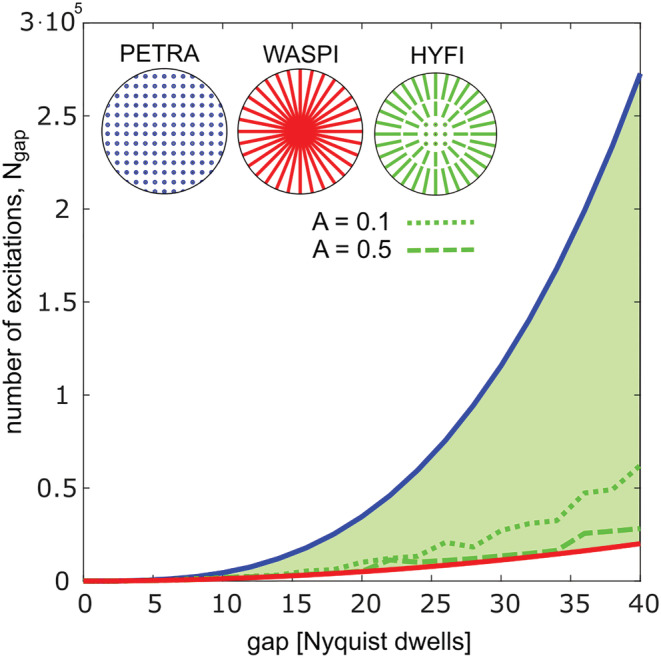
Number of excitations required to fill the complete inner k‐space by the different techniques. A decay with *T*_2_* = 100 dk is assumed. Circles at the top illustrate the acquisition geometries. The number of excitations *N*_*gap*_ required to fill the inner 3D k‐space evolves with $k_{\mathrm{gap}}^{3}$ for PETRA and $k_{\mathrm{gap}}^{2}$ for WASPI. In the proposed HYFI method, inner k‐space is filled by a combination of single‐point imaging (SPI) and radial acquisitions, and thus the green area enclosed by the curves for PETRA and WASPI becomes accessible. Green lines represent selected HYFI acquisitions with amplitude coefficients *A* = 0.1 and 0.5

Possible savings in scan time for the complete inner k‐space, including acquisition of both core and shells, are illustrated in Figure 5. To minimize scan time, in each radial shell the angular spoke density is adapted to fulfill the Nyquist criterion at the outer shell radius. In the limiting cases of PETRA and WASPI, the number of RF excitations *N*_*gap*_ required to fill a sphere of radius *k*_*gap*_ varies with the volume and the surface of the sphere, respectively. In HYFI, *N*_*gap*_ depends on the amplitude coefficient *A* and is bounded by these limits. As already suggested in Figure 4, small amplitude coefficients are sufficient to significantly decrease *N*_*gap*_.

The implementation of the described HYFI algorithm is governed by the discrete nature of sampling k‐space at Nyquist dwell distance. An example implementation is provided as Matlab source code at https://doi.org/10.3929/ethz‐b‐000415045.

### 2.2 Choice of imaging parameters

The parameters of all imaging experiments are listed in Tables 1 and S8.

**TABLE 1:** Parameters of all imaging experiments. The most important scan parameters are the target *T*_2_*, the bandwidth (BW), the field of view (FOV), the readout gradient (G) (used to encode the outer k‐space [c.f. Figure 1B]), the voxel size (Δ*r*), the matrix size (M), the dead time (Δ*t*), the k‐space gap (*k*_*gap*_), the number of signal averages (NSA), the pulse type, the pulse duration (Pul. dur), the coil type and the scanner field strength (B0). More details are provided in the supporting information

Sample	Target *T*_2_*	BW (kHz)	FOV (mm)	G (mT/m)	Δ*r* (mm)	M	Δ*t* (μs)	*k*_*gap*_ (dk)	NSA	Pulse	Pul. dur (μs)	Coil type	B0 (T)
Sphere simulations	100	1000	200	117.4	1	200	30	30	x	x	x	x	x
Stack of erasers	100	250	240	24.5	1.9	128	100	25	1	Block	2	Birdcage	7
Bone	200	766	90	199.9	0.4	256	40	31	8	Block	2	Surface	3
Head	200	2000	300	156.6	1.7	176	15	30	19	HSn	8	Birdcage	3
Knee	500	250	240	24.5	1.0	240	5.5	2	1	Block	2	Birdcage	7
Knee	500	250	240	24.5	1.0	240	200	50	1	Block	2	Birdcage	7
MnCl_2_ vials	55	852	100	199.9	1.0	100	55	47	1	HSn	10	Surface	3
MnCl_2_ vials	100	470	100	110.4	1.0	100	100	47	1	HSn	10	Surface	3
MnCl_2_ vials	200	235	100	55.2	1.0	100	200	47	1	HSn	10	Surface	3
MnCl_2_ vials	400	118	100	27.6	1.0	100	400	47	1	HSn	10	Surface	3
MnCl_2_ vials	600	78	100	18.3	1.0	100	600	47	1	HSn	10	Surface	3

In general, small dead times are desired to maximize signal amplitude and reduce scan duration. However, in some situations it may be preferable to deliberately extend the dead time and/or the gap size by inserting an additional delay before data acquisition. For example, short‐*T*_2_ components that cannot be resolved may be selectively suppressed in this way to improve image quality and quantification. Moreover, in the inner k‐space, data samples are acquired in a small time range bounded by the maximum shell acquisition duration *t*_*acq*_. When *t*_*acq*_ is small enough, this leads to similar *T*_2_ * weighting and chemical shift‐induced phase for all inner k‐space data. Consequently, increasing the inner k‐space volume to a substantial part of the support improves the accuracy of *T*_2_ * mapping based on a series of such data. In addition, it also reduces PSF blurring^23^ as well as chemical shift artifacts. In this work, increased dead time is employed for the suppression of components with extremely short *T*_2_, and reduction of chemical shift artifacts, as well as *T*_2_ * mapping.

When setting up a protocol, values for target *T*_2_* and amplitude coefficient *A* must be selected. A suitable choice of the parameter pair (*A*, *T*_2_*) is crucial for optimal HYFI performance. Figure 4 reveals that a good compromise between image quality and scan time is obtained for *A* ≲ 0.1. Indeed, for target *T*_2_* ≥ Δ*t*, the number of excitations required in the SPI region decreases by more than 95% while PSFs have negligible side lobes. However, in most cases the imaged samples contain multiple signal sources with different relaxation times *T*_2_*. Then a choice of *A* that is appropriate for a given *T*_2_* leads to stronger decay for signals with shorter *T*_2_* and hence potentially to artifacts. As usually not all relaxation times present in a sample are known a priori, an educated choice of the target *T*_2_* may not always be possible. As a rule of thumb, in the presence of multiple *T*_2_ * it is considered safe to choose a target *T*_2_ * = Δ*t* because signals with *T*_2_ * < Δ*t* will considerably decay before data acquisition. Moreover, as shown in the Results section, the target *T*_2_ * can be chosen to be larger than Δ*t* if the MR signal is dominated by sources with *T*_2_ * >> Δ*t*.

### 2.3 Hardware

All the experiments were performed on Achieva MRI systems (Philips Healthcare, Best, the Netherlands) at 3 or 7 T, complemented with symmetrically biased transmit‐receive switches^30^ with switching times of approximately 3 μs at 3 T and 1 μs at 7 T, custom‐made spectrometers^31^ with up to 4 MHz acquisition bandwidth and short digital filters with group delays down to 1.2 μs. Moreover, the 3 T scanner was equipped with a high‐performance gradient insert system capable of reaching 200 mT/m at full duty cycle^32^ and a broadband linear RF power amplifier BLA1000‐I E (Bruker Biospin, Wissembourg, France). Largely 1H‐free RF coils were used for both transmission and reception, a surface coil of 80 mm diameter and two birdcage coils.^33^, ^34^

Block and sweep hyperbolic secant (HSn) pulses^35^ with bandwidth matching the imaging bandwidth were used for excitation. HSn pulses were chosen for high bandwidth imaging, where block pulses posed too strong limitations on flip angles due to the combination of limited RF power and short durations. The pulse power was set empirically to obtain maximum signal in the tissues of interest. Flip angles were not calibrated but estimated to range from 2 to 4 degrees.

### 2.4 Samples

The relaxation constants of the samples were evaluated from mono‐ or double‐exponential fits on free induction decay (FID) signals measured at 3 T.

An imaging phantom with two different materials was created by placing a stack of erasers (Caran d'Ache 0149.340) with *T*_2_ * ≈ 380 μs onto a disk made of rubber with *T*_2_ * ≈ 130 μs.

A bone phantom was taken from a previous study.^22^ The piece of bovine tibia of 60 mm diameter and 25 mm thickness had been freed of sources of long‐lived MR signal (i.e. soft tissues). The signal relaxation appeared to be dominated by two *T*_2_ *s of about 10 and 150 μs. Hence, for imaging, a relatively long dead time of 40 μs was chosen deliberately to suppress the shorter component that cannot be resolved to the targeted submillimeter resolution and to focus on the longer‐*T*_2_ contributions, as well as to increase the PSF‐limited resolution.^23^

An imaging phantom with a range of *T*_2_ values was created by filling six solutions of MnCl_2_ with concentrations of 240, 120, 60, 30, 15 and 7.5 mM into glass vials. For measuring the transverse relaxation times, the solutions were filled into glass spheres of 20 mm diameter to minimize susceptibility effects. FID signals were fitted with single exponentials, providing decay constants of 54, 92, 181, 341, 663 and 1271 μs, respectively. For *T*_2_* mapping, a series of images was acquired with constant gap (*k*_*gap*_ = 47) and different dead times (Δ*t* = 55, 100, 200, 400 and 600 μs), where the gradient strength was adapted according to Table 1.

In vivo imaging of a knee and a head was conducted in healthy volunteers according to applicable ethics approval, and written informed consent was obtained from all subjects. For knee imaging, the dead time was intentionally increased to 200 μs to extend the inner k‐space and thus reduce chemical shift artifacts.

### 2.5 Image reconstruction and processing

Images were reconstructed using an iterative conjugate gradient algorithm,^36^ which in principle is capable of handling the complex density pattern of HYFI data alone, without the need to introduce specific merging filters. However, for improved convergence, density correction was applied as obtained by an iterative algorithm.^37^ Additionally, when modulated HSn pulses were used, RF pulse correction was performed.^38^ Finally, geometry and bias field corrections were applied to the head images to compensate for gradient nonlinearity^32^ and coil sensitivities.

For determining the SNR in images, additional noise data were acquired in the absence of RF excitation.^39^, ^40^ The average signal over a region of interest (ROI) in the magnitude sample image was divided by the standard deviation of the noise image in the same ROI. The SNR efficiency was obtained as ${\mathrm{SNR}}_{\mathrm{eff}}=\mathrm{SNR}/\sqrt{\text{scan time}}$, assuming the common relation of averaging and SNR. Finally, the relative scan time for equal SNR was calculated according to

(4) $$\tau_{\text{scan}}={\left(\frac{\mathrm{SN}R_{\mathrm{eff}}^{\text{PETRA}}}{\mathrm{SN}R_{\mathrm{eff}}^{\text{HYFI}}}\right)}^{2}.$$

More details about the SNR analysis are provided in the supporting information.

For interpreting the series of images acquired at different dead time Δ*t*, the echo time is defined as TE = Δ*t* for both PETRA and HYFI, according to the convention that TE indicates the time at which the k‐space center is acquired. *T*_2_* fitting was performed with single‐exponential functions and all amplitudes were normalized with the respective value obtained at t = 0.

### 2.6 Simulations

Simulations were performed to evaluate artifacts due to *T*_2_* decay in relation to improvements in scan efficiency, as a basis for optimizing the HYFI parameter choice. 1D PSFs were calculated by Fourier transforming MTFs. For 3D simulations, the k‐space signal of spheres with 50, 30, 18 and 9 mm diameter and *T*_2_ of 100 μs was created analytically,^41^ assuming identical magnetization after each excitation. Images were reconstructed with the algorithm described above by following the same pipeline as for the experimental data. Corresponding imaging parameters are given in Table 1.

## 3 RESULTS

**FIGURE 6:**
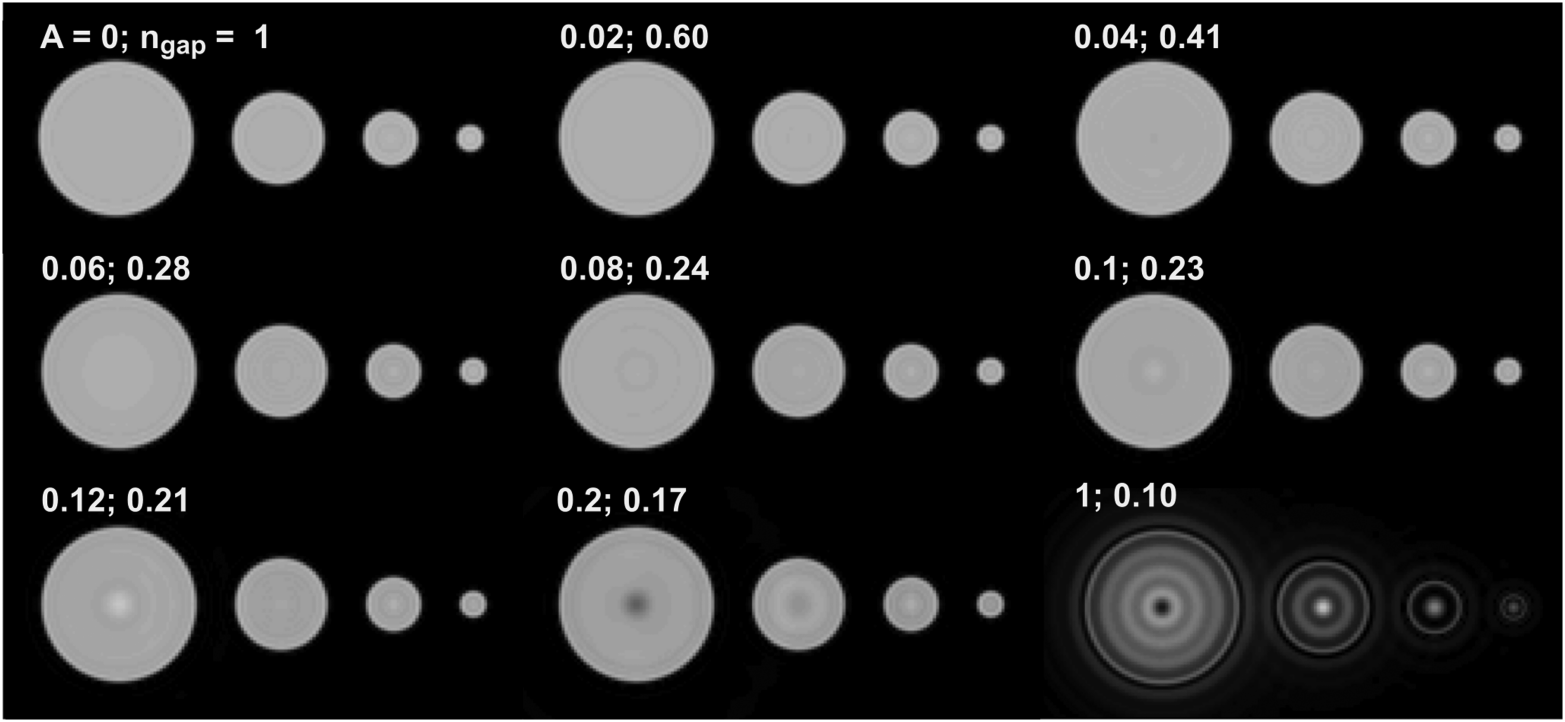
3D HYFI simulations illustrating the effect of the amplitude coefficient *A* on image quality and scan efficiency. Imaging of spheres with 50, 30, 18 and 9 mm diameter was simulated assuming *T*_2_ = 100 μs and *k*_*gap*_ = 30 dk. Other parameters are listed in Table 1 and Table S8 in the supporting information. Savings in scan time are quantified with *n*_*gap*_, the number of excitations needed to acquire the inner k‐space relative to the case *A* = 0. As *A* increases from 0 (PETRA) to 1 (WASPI), *k*_*SPI*_ decreases and *n*_*gap*_ decreases. The latter quickly reaches 28% (*A* = 0.06) with preserved image quality. Above this value, *n*_*gap*_ decreases slower and artifacts start to appear in the center of the larger spheres, suggesting that for such samples the optimal amplitude coefficient *A* resides below 0.1. Image profiles of a few representative cases are shown in Figure S2

The effect of the amplitude coefficient *A* on image quality and scan efficiency is illustrated with 3D simulations (Figure 6). Corresponding image profiles are shown in Figure S2. As the amplitude coefficient *A* increases, the relative number of excitations required to fill the gap, *n*_*gap*_, decreases, thus increasing the scan efficiency. For this sample, images without noticeable artifacts are obtained with *A* less than 0.1.

**FIGURE 7:**
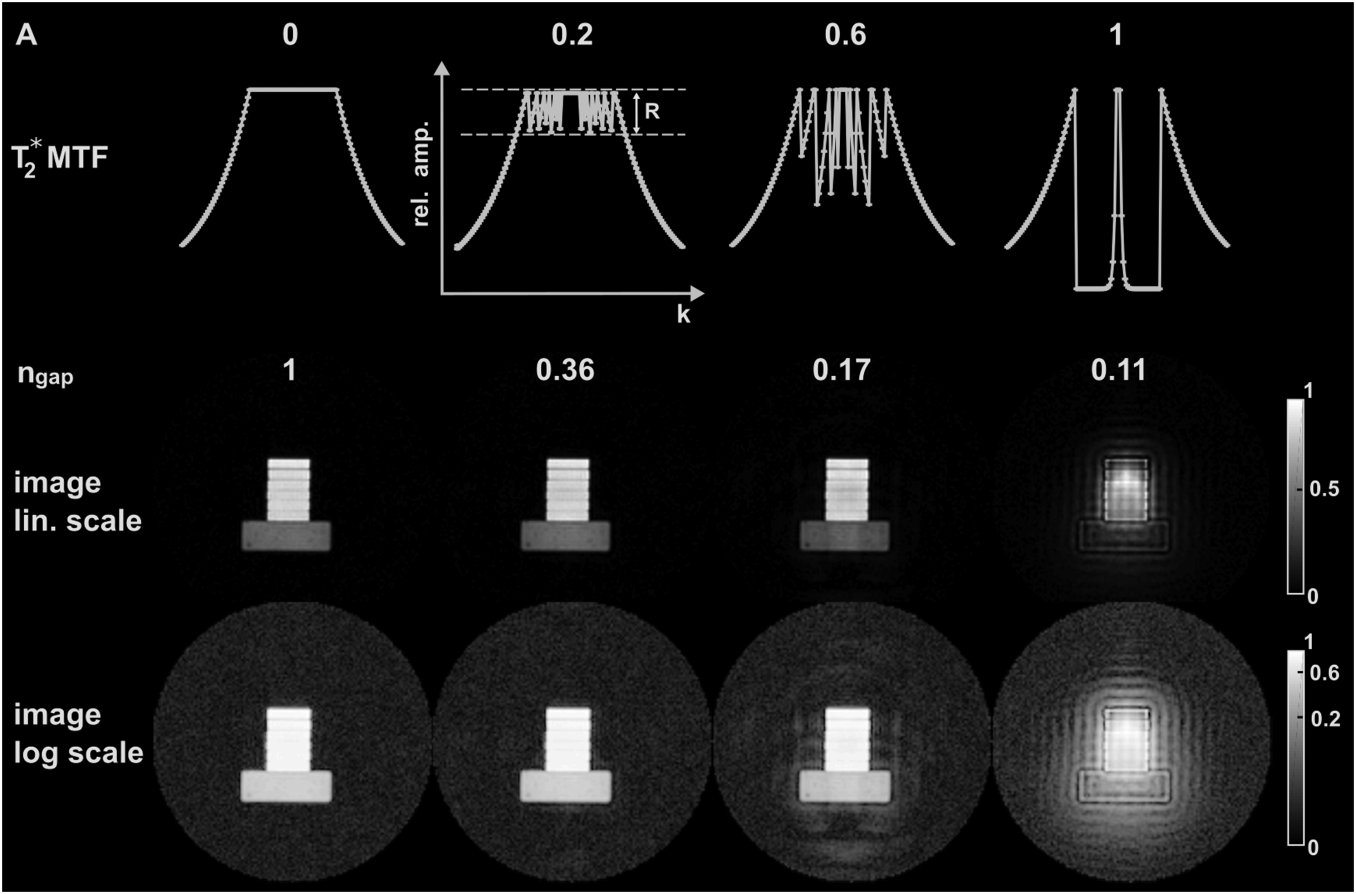
Demonstration of the HYFI principle in a phantom experiment. The first row represents the amplitude coefficient *A* growing from 0 (PETRA) to 1 (WASPI) with intermediate values corresponding to HYFI. Underneath are the associated 1D k‐space *T*_2_*‐related modulation transfer functions (MTFs) (c.f. Figure 3) and the relative number of excitations required to fill the gap, *n*_*gap*_. The resulting images are shown in the two bottom rows with linear (lin.) and logarithmic (log) grayscales. They illustrate the importance of the parameter selection in HYFI. Indeed, at small *A*, the scan time is reduced and image quality is preserved, while at large *A*, the strong *T*_2_* weighting in the gap leads to image artifacts

The phantom experiment in Figure 7 demonstrates the HYFI principle over a large range of *A*. For *A* = 0, the inner k‐space is acquired in an SPI fashion, leading there to a constant plateau of *T*_2_ * weighting, maximum *n*_*gap*_ and artifact‐free images. When *A* increases, radial shells with restricted decay replace part of the SPI plateau and *n*_*gap*_ diminishes. However, this also creates progressively increasing irregularities in the MTF, which in turn leads to increasingly large ringing artifacts out and inside the imaged object.

**FIGURE 8:**
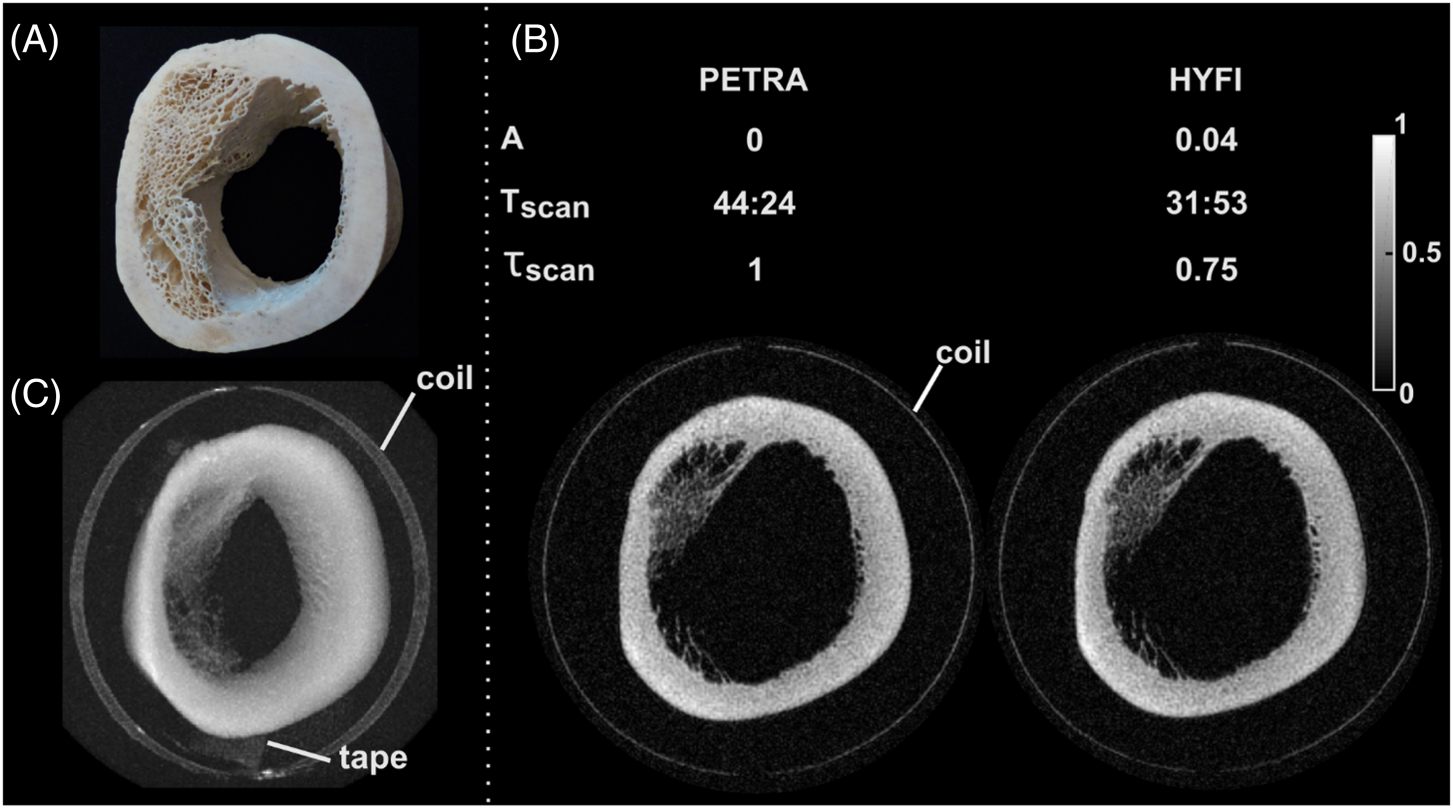
Bone imaging. (A) Picture of the imaged segment of a bovine tibia. (B) Comparison of PETRA and HYFI, with amplitude coefficient *A*, total scan time T_scan_ (min:s), relative total scan time for the same SNR, *τ*_*scan*_ (including both inner and outer k‐space), and the images acquired with Δ*t* = 40 μs. They show virtually identical quality but the HYFI scan is considerably faster. (C) Maximum intensity projection of the HYFI image demonstrating high image quality over the whole field of view. The glue fixing the copper coil to the glass support and the tape holding the sample to the bed are also depicted. This figure illustrates that HYFI allows substantial increase in scan efficiency compared with PETRA for high‐resolution imaging of tissues with *T*_2_s of hundreds of microseconds, such as in bone in case large gaps are involved. Figure 8A is reproduced from Froidevaux et al.^23^ with permission

In Figure 8, the performance of PETRA and HYFI are compared for imaging a sample of bovine tibia. Both techniques lead to high quality images depicting fine trabecular bone structure. Other details appear in the maximum intensity projection, such as glue from the coil conductor and the fixation tape. In HYFI, the choice of a small *A* = 0.04 is sufficient to significantly improve the SNR efficiency compared with PETRA, which translates into a 25% decrease of total scan time for the same SNR.

**FIGURE 9:**
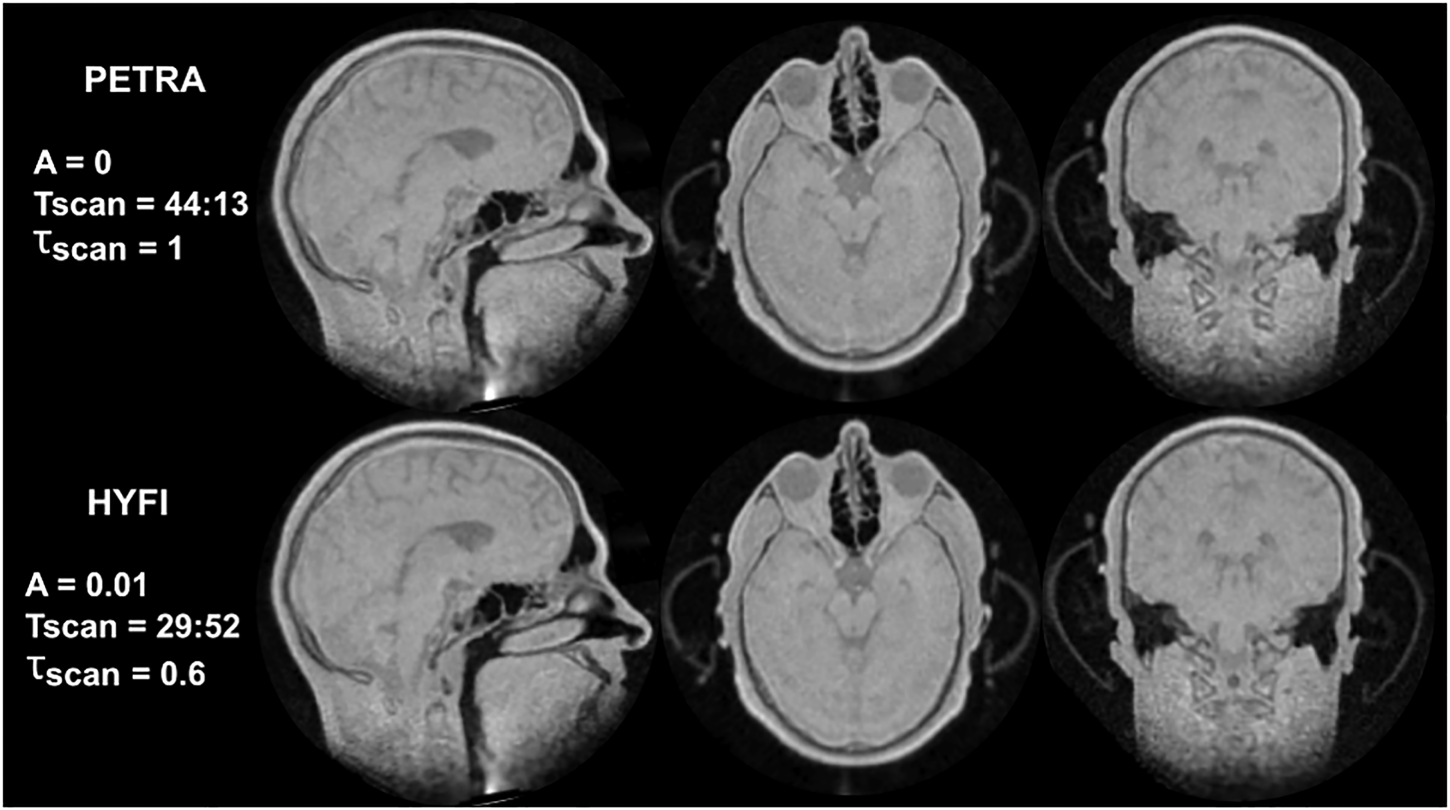
High gradient head imaging. The displayed parameters are amplitude coefficient *A*, total scan time T_scan_ (min:s) and relative scan time for the same SNR, *τ*_*scan*_. Top row: three perpendicular slices of the same 3D volume acquired with PETRA. Bottom row: the corresponding HYFI images are of comparable quality but *τ*_*scan*_ is considerably reduced. Thanks to the short dead time (Δ*t* = 15 μs) and the high bandwidth (2 MHz), these proton density‐weighted images contain and resolve signals from short‐*T*_2_ materials and tissues, such as the plastic cover of the ear protection helmet, teeth, skull and other bones, and possibly myelin in the brain. Furthermore, high robustness against local susceptibility gradients is observed in the sinuses. The higher scan efficiency of HYFI as well as reduced acoustic noise considerably improve patient comfort during such a long scan. Note that the artifact in the neck region (first column) is created by signal stemming from the chest area, which is aliased into the field of view due to gradient ambiguity

The head images in Figure 9 were acquired with an unusually high bandwidth of 2 MHz and confirm the above results. The SNR efficiency of HYFI is enhanced compared with PETRA and leads to a 40% scan time reduction for the same SNR while preserving image quality.

**FIGURE 10:**
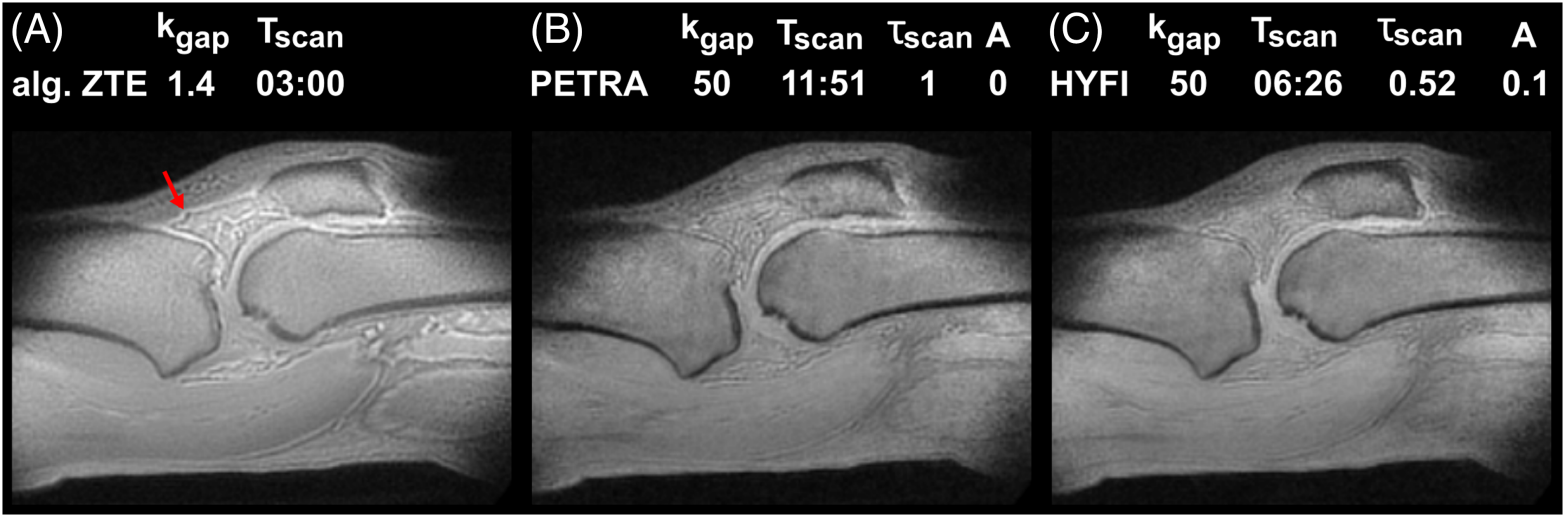
Knee imaging at 7 T. (A) Algebraic (alg.) zero echo time (ZTE) imaging with short dead time (Δ*t* = 5.5 μs) and hence small gap, short scan time, but relatively strong chemical shift artefacts at water‐fat boundaries, as indicated by the red arrow. (B) PETRA image with the gap intentionally increased to 50 dk (Δ*t* = 200 μs) to acquire most of the data in a single‐point imaging (SPI) manner and hence decrease chemical‐shift artefacts. (C) Same as in (B), except that the inner k‐space is filled with HYFI (*A* = 0.1). In this case the relative scan time for the same SNR (*τ*_*scan*_) decreases by 48% while image quality is preserved. Each image is an average of three in‐plane slices leading to an effective resolution of 1 x 1 x 3 mm^3^

Figure 10 shows the influence of the dead‐time gap on chemical shift artifacts in ZTE knee imaging. At minimum dead time Δ*t* = 5.5 μs leading to *k*_*gap*_= 1.4 dk, signal intensity is maximized and, moreover, the missing data points can be reconstructed algebraically leading to minimum scan duration. However, due to signal dephasing during the spoke sampling of 480 μs, chemical shift artefacts appear at water‐fat boundaries.^17^ Increasing the dead time to 200 μs enlarges the inner k‐space to 50 dk radius and thus reduces the acquisition time range for data located in this region. In PETRA (Figure 10B), signal can dephase only within 1 dk of 4 μs. Hence, while accepting a loss of signal intensity associated with the longer dead time, chemical shift artifacts are strongly reduced and the resolution at water‐fat interfaces is improved. However, scan time is substantially increased. Under the same circumstances, HYFI provides similar image quality but reduces the acquisition duration for the same SNR by 48% compared with PETRA (Figure 10C).

**FIGURE 11:**
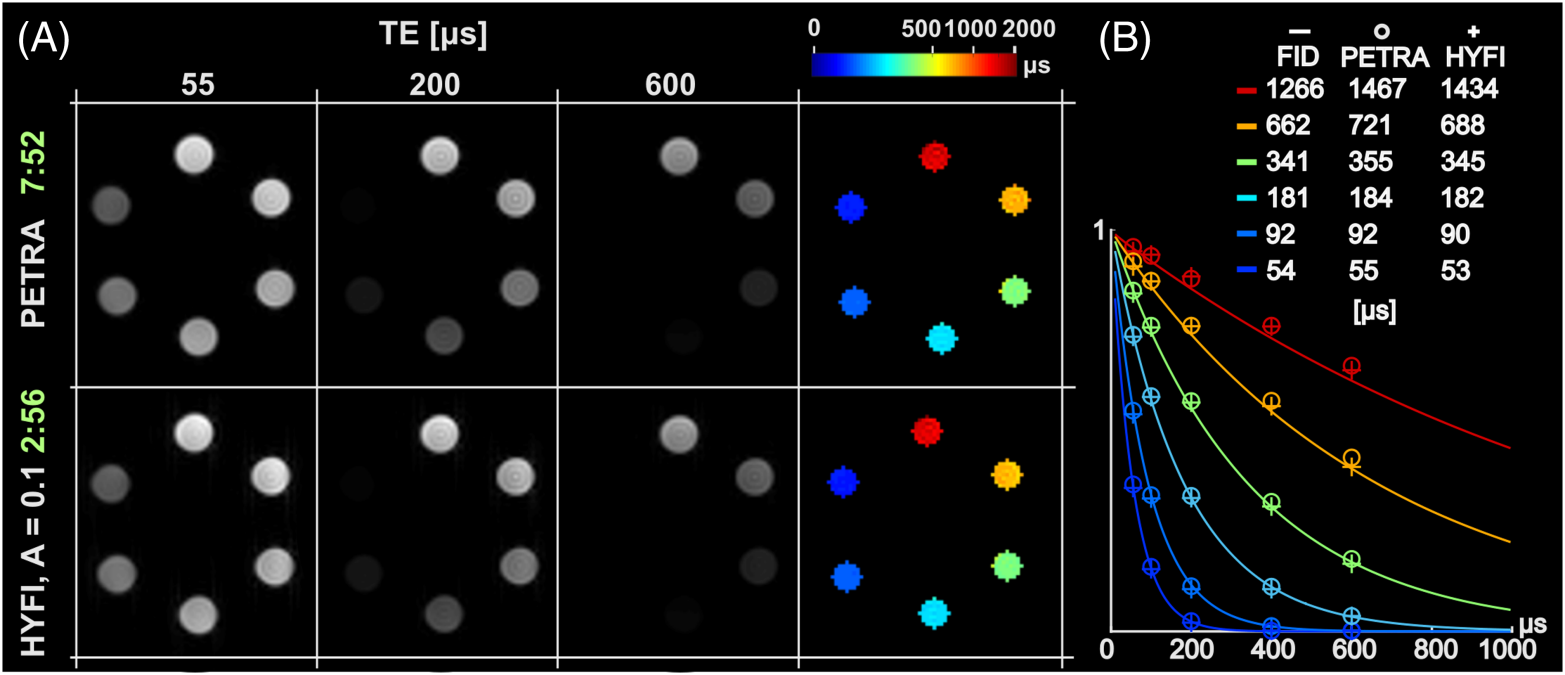
*T*_2_* mapping in MnCl_2_ solutions with rapid transverse relaxation. (A) Series of PETRA and HYFI images taken at different echo time *TE* = ∆*t* = 55, 100, 200, 400 and 600 μs (only a subset is displayed). Maps of transverse relaxation times were obtained by pixel‐wise fitting the signal decay (last column). The scan time of individual images is given in min:s (green), which shows a considerable reduction for HYFI. (B) The relative intensities of the signal averaged over each sample are plotted as a function of *TE* (circles and crosses) and overlaid with the free induction decay (FID) measured in each MnCl_2_ solution (solid line). Fitted single‐exponential *T*_2_* values are given in the legend. These results demonstrate that *T*_2_* of very rapidly decaying signal can be mapped efficiently with the HYFI technique

*T*_2_ * mapping of short‐*T*_2_ samples is demonstrated in Figure 11. To enable accurate fitting, the inner k‐space was deliberately increased up to the outer limit of the support such that the whole k‐space was acquired within a restricted time range. In this way, PETRA approaches pure SPI acquisition.^26^ Figure 11A shows that at TE = 55 μs, all the vials are well depicted and image intensity drops at larger TE in the short‐*T*_2_ samples. For the same image and *T*_2_ * map quality, the scan time of HYFI is 62% lower than PETRA. Figure 11B shows a good correspondence between the fit of average map intensities and the fit of the FID, especially in the short‐*T*_2_ * range. Mean values including the 95% confidence interval are given in Table S7 in the supporting information. Two observations can be made: (a) there is an increasing divergence between *T*_2_* values fitted from FIDs and images as *T*_2_ * gets larger, and (b) the relaxation times fitted from the HYFI data are slightly but consistently smaller than the PETRA results. The first observation is assigned to residual B0 inhomogeneity in the samples used for FID measurements, leading to smaller effective *T*_2_* values. The second effect results from the fact that the echo time TE is considered equal to the dead time ∆*t*, which fits PETRA better than HYFI. Indeed, in HYFI, signal is still acquired for a duration *t*_*acq*_ after ∆*t*. During that time, the signal decays and appears smaller than in PETRA. Also, as the target *T*_2_* (and hence *t*_*acq*_) increases with TE (c.f. Table 1), this effect also increases with TE and thus leads to smaller fitted *T*_2_ * values.

## 4 DISCUSSION

HYFI, a recently introduced ZTE‐based method with hybrid filling of the inner k‐space, was described in detail and its performance in the presence of large k‐space gaps was studied. It was demonstrated that with HYFI, substantial reductions in scan time can be enabled while preserving image quality compared with the PETRA technique. The advantage of HYFI increases with gap size and is therefore of particular interest at high imaging bandwidth, large minimum RF dead times, and large dead times selected to manipulate image contrast. The technique was successfully employed for imaging on different phantoms as well as in vivo and ex vivo.

Using HYFI with high efficiency and fidelity necessitates a suitable choice of the parameter pair (*A*, *T*_2_*). The simulations in Figure 6 demonstrate that in the presence of a single *T*_2_, *A* ≲ 0.1 provides considerable savings in scan time at still high image quality. However, as *A* is increased, artifacts become more likely due to coherent interaction of increasingly large PSF side lobes occurring predominantly in the center of large objects.

In more common cases involving different tissues and molecules and thus a range of transverse relaxation times, the choice of the target *T*_2_* requires extra considerations. Selecting the smallest *T*_2_* present in the sample is safe but usually too conservative. Typically, components with *T*_2_* less than Δ*t* have little influence on the final image and choosing a target *T*_2_* = Δ*t* can be considered an appropriate rule of thumb (Figure 11). Moreover, the target *T*_2_* might be increased to values larger than Δ*t* without degrading image quality if the signal is dominated by components with longer *T*_2_*. In the presented in vivo data, target *T*_2_* of a few hundred microseconds still lead to images without noticeable artifacts (Figures 9 and 10), although components with clearly faster relaxation but lower intensity (e.g. myelin or collagen) are present. Only if the latter signals should be extracted from the data, they need to be considered for setting the target *T*_2_*.

In the performed experiments, the reductions in scan time of HYFI with respect to PETRA range from 25% to 62% (Figures 8, 9, 10, 11). The influencing factors are gap size, spatial resolution and the parameter pair (*A*, *T*_2_*). The relative number of excitations required to fill the inner k‐space with HYFI decreases with increasing gap size compared with PETRA (Figure 5). This explains why the best HYFI performance is obtained in the examples shown in Figures 10 and 11, where gaps were deliberately increased to high values to reduce chemical shift artefacts and perform *T*_2_* mapping, respectively. The resolution determines the time spent for the acquisition of the outer k‐space, which in turn affects the relative scan time spent on the inner k‐space. Thus, as resolution and hence scan time are increased, the absolute time difference between PETRA and HYFI does not change but the relative advantage of HYFI diminishes. Finally, the selection of the parameter pair (*A* , *T*_2_*) influences the k‐space trajectory and affects both scan time and image SNR. As the amplitude coefficient *A* increases, the SPI region decreases and is replaced by radial acquisitions, which leads to higher k‐space data density and thus a reduction of image noise variance.^23^ However, the data points experience stronger *T*_2_* weighting (Figure 3), which translates into a smaller integral of the MTF and hence to a smaller PSF main lobe (Figure 4), leading to decreased voxel intensity. These two effects partly compensate each other. For small amplitude coefficients they even largely balance and the improvement of HYFI in SNR efficiency can be well approximated as if arising from scan time reduction alone. High‐resolution imaging of short‐*T*_2_ components benefits from the use of high gradients.^42^ As shown in Figure 8, bone tissue with *T*_2_* ≈ 150 μs can be imaged at an isotropic resolution of 400 μm using a gradient strength of 200 mT/m. Such high gradients induce high bandwidths and thus large k‐space gaps, especially in large FOVs as required for imaging in humans (Figure 9). In such circumstances, substituting a large part of the SPI region by radial spokes with HYFI particularly improves scanning efficiency. Moreover, at large gradient amplitude Cartesian SPI acquisition can produce significant mechanical vibrations and acoustic noise due to partly large gradient switching between k‐space directions. With HYFI, as long as the gradient can be used at full duty cycle (i.e. without switching back to zero amplitude), these effects are significantly reduced because k‐space directions are uniformly distributed in all directions and sequentially accessed along spiral spoke ordering^43^ requiring slower gradient switching, thus clearly improving patient comfort.

The results of this work indicate the potential of HYFI for direct imaging of ultrashort‐*T*_2_ components such as in the myelin bilayer in the brain. However, as observable in Figure 9, basic ZTE sequences lead to mostly proton density‐weighted images, and some kind of selectivity is required to isolate the tissues of interest. One possibility to achieve *T*_2_* selection uses postprocessing of a series of images acquired after different dead times. An example of such an experiment is shown in Figure 11, where *T*_2_* mapping of MnCl_2_ solutions was performed by fitting exponential signal decays. This kind of approach has been shown to enable *T*_2_* selection of ultrafast relaxing MR signals in the brain that can potentially be assigned to the myelin bilayer.^10^ Further improvements in quantification with HYFI‐based *T*_2_* mapping are expected with a more advanced definition of TE or signal models taking into account the precise sequence timing.

Clinical scanners with state‐of‐the‐art hardware specifications (e.g. an RF switching time of approximately 30 μs, ^44^ gradient slew rate of approximately 200 mT/m and maximum gradient strength of approximately 80 mT/m, yet at limited duty cycle^17^) may be used for short‐*T*_2_ PETRA imaging and will lead to gap sizes of about 30–40 dk. Thus, assuming a TR of a few milliseconds, the acquisition of the inner k‐space takes several minutes (c.f. Figure 4). In such a situation and as illustrated in this paper, substantial improvement in SNR efficiency can be expected when using HYFI instead of PETRA. If the same scanners are used with lower bandwidth (e.g. G < 40 mT/m), the advantage of HYFI is limited to lower acoustic noise and reduced mechanical vibrations. In the special case of a combination of low gradients and large dead times (e.g. G = 10 mT/m, RF switching time of approximately 50 μs), the dead time could be used to ramp up the gradient to its target strength, thus allowing smaller excitation bandwidths at reduced gap sizes and avoiding the need for HYFI, yet at the price of reduced spatial resolution.^23^ A similar idea was exposed in the ramped hybrid encoding (RHE) technique,^45^ where the readout gradient is lowered during RF excitation and increased to full strength afterwards during data acquisition. In such cases, k‐space calibration is required because timing errors and eddy current effects distort the k‐space trajectory. Other alternatives to PETRA and hence HYFI are SWIFT^46^ and cSWIFT,^47^ where gaps are very small or inexistent, respectively. However, the first one comes at an SNR penalty and limited bandwidth^48^ and the second approach is particularly sensitive to RF coil loading variations.

Finally, there are situations where a large part of the data should be acquired within a small time range as required for chemical shift artifact reduction or *T*_2_* mapping. For example, for performing the latter, an SPI acquisition of the whole k‐space support is favorable, as shown in Figure 11. Note that in this context, gradients should be switched on before the RF pulse because large gaps are actually targeted. Hence, even with clinical scanners, HYFI can be considered an efficient alternative to SPI methods^26^, ^49^ in situations where creating gradient echoes is hampered by limited gradient performance.

## 5 CONCLUSION

The HYFI technique provides both high SNR efficiency and image quality, thus outperforming previously known ZTE‐based pulse sequences. It is particularly advantageous in situations involving large dead times or high gradient strengths where PETRA suffers from long and noisy SPI acquisitions. Promising applications include direct imaging of ultrashort *T*_*2*_ components, such as the myelin bilayer or collagen, *T*_2_* mapping of ultrafast relaxing signals, and ZTE imaging with reduced chemical shift artifacts.

## Supporting information


**Figure S1** 1D point spread functions (PSFs) for varying amplitude coefficient A. Simulations were performed for a matrix size of 200 pixels with T_2_* = 100 dk and k_gap_ = 30 dk. For small values of A, the PSFs are very similar. However, the size of the side lobes quickly increases as A approaches 1.
**Figure S2** Profiles of the 3D simulations shown in Figure 4 demonstrating the effects of the decay coefficient A on image quality. Each profile is taken horizontally on the middle line of 4 selected images (A = 0, 0.04, 0.1, 0.2). As A increases, the overall image intensity decreases (by a factor smaller than A) and artifacts start to appear in the center of each object, especially in the largest one. Noticeably, the sharpness of the edges is hardly affected. All of these effects can be understood by looking at the HYFI MTF (Figure 3 of the main paper. First, as long as the overall PSF shape remains close to a delta function, the image intensity is related to the PSF maximum and hence to the integral of the MTF. As A increases, the MTF decreases by a factor A only in the inner k‐space at the end of each radial shell. Thus, its integral and hence the image intensity decreases, but by a factor smaller than A. Second, the irregularities in the MTF arise around the k‐space center and not on the edges of the k‐space support (as opposed to usual Gibbs ringing). Hence, the related artifacts are expressed as low frequency modulation appearing essentially at the center of the larger objects where PSFs of surrounding pixels can constructively interfere. However, the high‐frequency part of the object and hence the resolution of the edges remain mostly unchanged.
**Table S1** Images used by methods 1 and 2 for SNR calculations. Only one representative slice is shown here but calculations were done in 3 dimensions. In method 2, the ROIs as well as corresponding averages and standard deviation are shown in color.
**Table S2** Absolute and relative values calculated with methods 1 and 2.
**Table S3** Images used by methods 1 and 2 for SNR calculations. In method 1, the mask position is represented over the magnitude image with transparency. In methods 2, the ROIs as well as corresponding averages and standard deviation are shown in colors.
**Table S4** Absolute and relative values calculated with methods 1 and 2.
**Table S5** Images used by methods 1 and 2 for SNR calculations. Only one representative slice is shown here but calculations were done in 3 dimensions. In method 1, the mask was calculated by applying a threshold to the magnitude image. In method 2, the ROIs as well as corresponding averages and standard deviation are shown in color.
**Table S6** Absolute and relative values calculated with methods 1 and 2.
**Table S7** Results of *T*_2_* fitting. *T*_2_* values [μs] of 6 MnCl_2_ solutions were fitted with single exponential functions on data measured with FIDs, PETRA and HYFI. Results include the 95% confidence interval.
**Table S8** Detailed parameter table with the amplitude coefficient *A*, the target *T*_2_*, the shell acquisition time t_acq_, the number of radial shells in inner k‐space, the number of spokes in inner k‐space, the number of spokes in outer k‐space, the gap in Nyquist dwells, the SPI core radius in Nyquist dwells, the number of excitations required in the SPI core N_SPI_, the repetition time TR, the number of sampling averages NSA and the total scan time.

## Data Availability

The Matlab files that support the findings of this study are openly available in the ETH research collection in “Supporting material for HYFI paper” at https://doi.org/10.3929/ethz‐b‐000415045.

## ENDNOTES

* In the tissues targeted in this work, short *T*_2_ largely governs *T*_2_ ***. Therefore, to describe tissue properties and signal relaxation in general, the term *T*_2_ is used, whereas *T*_2_ *** is employed to characterize the decay in actual signal acquisition.

† Throughout this manuscript, the term “data points” corresponds to k‐space samples separated by Nyquist distance, dk = 1/FOV.

‡
The exact number of points cannot be calculated analytically. It corresponds to the number of nodes of a Cartesian grid which have a radial distance to the k‐space center smaller than kgap. However, a very good approximation can be found by multiplying the volume of the gap (given by the above formula) with the density of data points acquired on the Cartesian grid, (which in this case is 1 data point per dk3). In order to obtain an integer number, kgap may be rounded to the closest integer value.
