# Supplementary material for: HYFI: Hybrid filling of the dead‐time gap for faster zero echo time imaging
Source: NMR Biomed. 2021 Feb 23;34(6):e4493. doi: 10.1002/nbm.4493 (PMC8244056; doi:10.1002/nbm.4493)
Supplement: Supplementary file 1 — Figure S1 1D point spread functions (PSFs) for varying amplitude coefficient A. Simulations were performed for a matrix size of 200 pixels with T2* = 100 dk and kgap = 30 dk. For small values of A, the PSFs are very similar. However, the size of the side lobes quickly increases as A approaches 1. Figure S2 Profiles of the 3D simulations shown in Figure 4 demonstrating the effects of the decay coefficient A on image quality. Each profile is taken horizontally on the middle line of 4 selected images (A = 0, 0.04, 0.1, 0.2). As A increases, the overall image intensity decreases (by a factor smaller than A) and artifacts start to appear in the center of each object, especially in the largest one. Noticeably, the sharpness of the edges is hardly affected. All of these effects can be understood by looking at the HYFI MTF (Figure 3 of the main paper. First, as long as the overall PSF shape remains close to a delta function, the image intensity is related to the PSF maximum and hence to the integral of the MTF. As A increases, the MTF decreases by a factor A only in the inner k‐space at the end of each radial shell. Thus, its integral and hence the image intensity decreases, but by a factor smaller than A. Second, the irregularities in the MTF arise around the k‐space center and not on the edges of the k‐space support (as opposed to usual Gibbs ringing). Hence, the related artifacts are expressed as low frequency modulation appearing essentially at the center of the larger objects where PSFs of surrounding pixels can constructively interfere. However, the high‐frequency part of the object and hence the resolution of the edges remain mostly unchanged. Table S1 Images used by methods 1 and 2 for SNR calculations. Only one representative slice is shown here but calculations were done in 3 dimensions. In method 2, the ROIs as well as corresponding averages and standard deviation are shown in color. Table S2 Absolute and relative values calculated with methods 1 and 2. Table S3 [file NBM-34-e4493-s001.docx]

# Supporting information for “HYFI: Hybrid filling of the dead-time gap for faster zero echo time imaging” by R. Froidevaux et al.


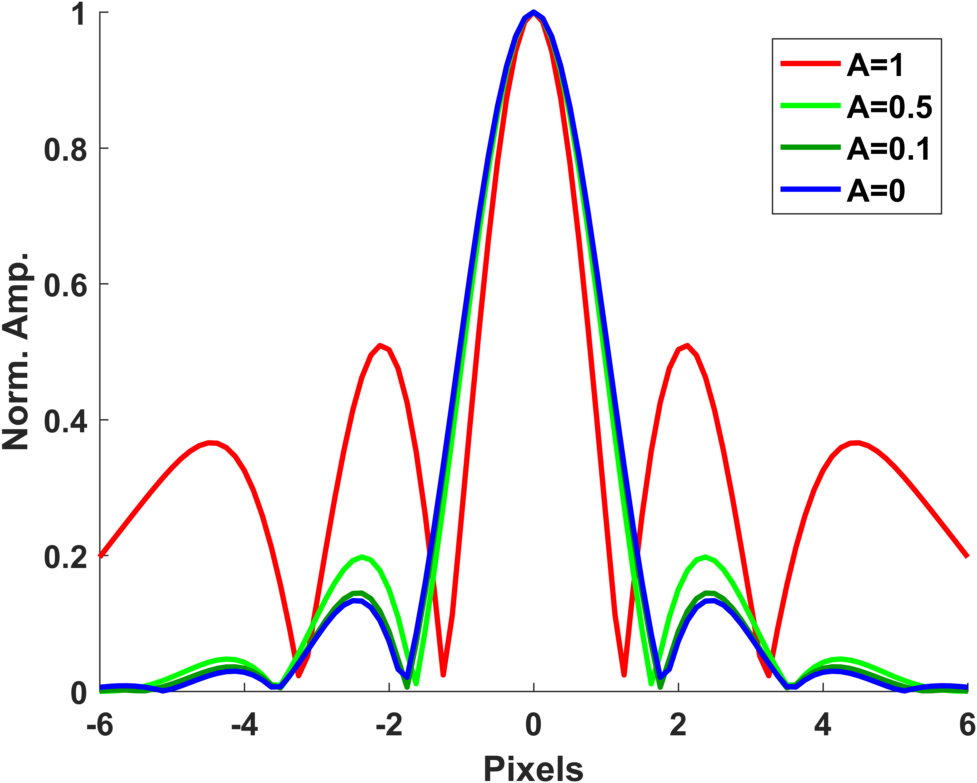


Figure S1: 1D point spread functions (PSFs) for varying amplitude coefficient A. Simulations were performed for a matrix size of 200 pixels with T_2_* = 100 dk and k_gap_ = 30 dk. For small values of A, the PSFs are very similar. However, the size of the side lobes quickly increases as A approaches 1.


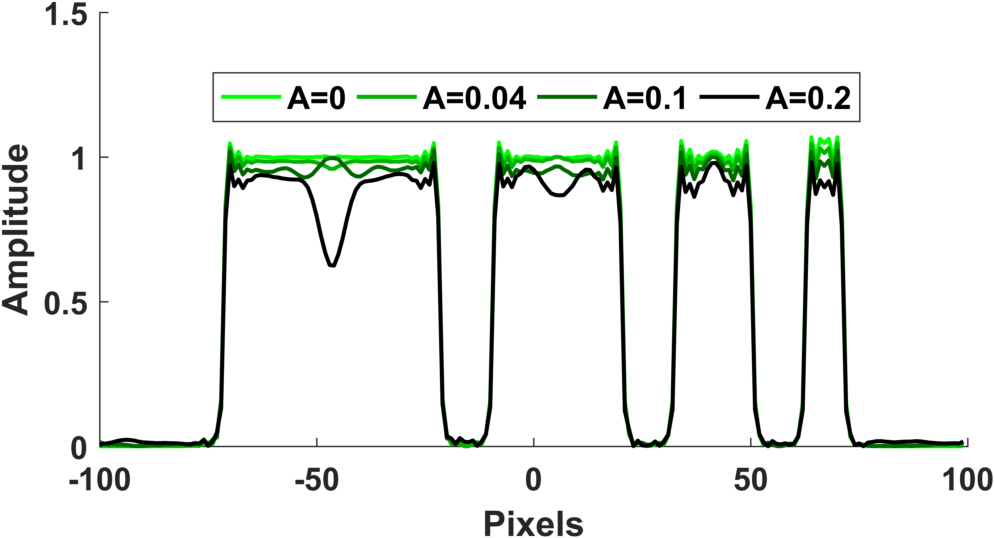


Figure S2: Profiles of the 3D simulations shown in Fig. 4 demonstrating the effects of the decay coefficient A on image quality. Each profile is taken horizontally on the middle line of 4 selected images (A = 0, 0.04, 0.1, 0.2). As A increases, the overall image intensity decreases (by a factor smaller than A) and artifacts start to appear in the center of each object, especially in the largest one. Noticeably, the sharpness of the edges is hardly affected. All of these effects can be understood by looking at the HYFI MTF (Fig. 3 of the main paper. First, as long as the overall PSF shape remains close to a delta function, the image intensity is related to the PSF maximum and hence to the integral of the MTF. As A increases, the MTF decreases by a factor A only in the inner k-space at the end of each radial shell. Thus, its integral and hence the image intensity decreases, but by a factor smaller than A. Second, the irregularities in the MTF arise around the k-space center and not on the edges of the k-space support (as opposed to usual Gibbs ringing). Hence, the related artifacts are expressed as low frequency modulation appearing essentially at the center of the larger objects where PSFs of surrounding pixels can constructively interfere. However, the high-frequency part of the object and hence the resolution of the edges remain mostly unchanged.

## SNR calculations

This section provides details about SNR calculations and compares different methods:

- Method 1: The signal is calculated by averaging the magnitude image over a region of interest (ROI) defined by a mask. The image noise is reconstructed from a noise-only dataset using the same algorithm as used for image reconstruction. The noise standard deviation is then measured over the same ROI as used in the magnitude image (the mask).
- Method 2: Signal and noise standard deviation are measured from different ROIs of the same magnitude image (represented by violet and yellow circles, respectively).

Absolute and relative results are given for 3 datasets used in this paper (bone, head and knee). The SNR calculation methods are illustrated with single slices of 3D PETRA images.

### Bone sample

| Method | Mask | Magnitude image | Noise image |
| --- | --- | --- | --- |
| 1 |  |  |  |
| 2 |  |  |  |

Table S1: Images used by methods 1 and 2 for SNR calculations. Only one representative slice is shown here but calculations were done in 3 dimensions. In method 2, the ROIs as well as corresponding averages and standard deviation are shown in color.

|  | Scan time  [s] | SNR1 | SNR2 | SNReff1  [$s^{-1/2}$] | SNReff2  [$s^{-1/2}$] |
| --- | --- | --- | --- | --- | --- |
| HYFI | 1913 | 16.4 | 19.2 | 0.375 | 0.439 |
| PETRA | 2665 | 17.0 | 19.4 | 0.330 | 0.376 |
| Ratio (HYFI/PETRA) | 0.72 | 0.96 | 0.99 | 1.14 | 1.17 |

Table S2: Absolute and relative values calculated with methods 1 and 2.

### Head

After reconstruction of raw images, non-linearity and bias field corrections were applied in the same way for both PETRA and HYFI images for display in Figure 9. These corrections introduce local intensity variations in the field of view that are independent on the acquisition methods. For this reason and in order to calculate the method-related SNR, calculations of SNR were done on the raw images as illustrated below. The artifacts on the edges of the FOV are assigned to aliasing of signals located outside of the field of view.

| Method | Mask | Magnitude image | Noise image |
| --- | --- | --- | --- |
| 1 |  |  |  |
| 2 |  |  |  |

Table S3: Images used by methods 1 and 2 for SNR calculations. In method 1, the mask position is represented over the magnitude image with transparency. In methods 2, the ROIs as well as corresponding averages and standard deviation are shown in colors.

|  | Scan time [s] | SNR1 | SNR2 | SNReff1 [$s^{-1/2}$] | SNReff2  [$s^{-1/2}$] |
| --- | --- | --- | --- | --- | --- |
| HYFI | 1792 | 49.46 | 46.90 | 1.17 | 1.11 |
| PETRA | 2632 | 46.72 | 43.30 | 0.91 | 0.84 |
| Ratio (HYFI/PETRA) | 0.68 | 1.06 | 1.08 | 1.28 | 1.31 |

Table S4: Absolute and relative values calculated with methods 1 and 2.

### Knee

In order to improve image quality, 3 slices were averaged for display in Fig. 10. However, the calculation of SNR was done on raw images, without averaging.

| Method | Mask | Magnitude image | Noise image |
| --- | --- | --- | --- |
| 1 |  |  |  |
| 2 |  |  |  |

Table S5: Images used by methods 1 and 2 for SNR calculations. Only one representative slice is shown here but calculations were done in 3 dimensions. In method 1, the mask was calculated by applying a threshold to the magnitude image. In method 2, the ROIs as well as corresponding averages and standard deviation are shown in color.

|  | Scan time [s] | SNR1 | SNR2 | SNReff1 [$s^{-1/2}$] | SNReff2 [$s^{-1/2}$] |
| --- | --- | --- | --- | --- | --- |
| HYFI | 386 | 15.66 | 22.10 | 0.80 | 1.12 |
| PETRA | 711 | 15.31 | 21.30 | 0.57 | 0.80 |
| Ratio (HYFI/PETRA) | 0.54 | 1.02 | 1.04 | 1.39 | 1.41 |

Table S6: Absolute and relative values calculated with methods 1 and 2.

## *T_2_** fitting

| Vial | FID | PETRA | HYFI |
| --- | --- | --- | --- |
| 1 | 1266.8 (1266.4, 1267.1) | 1467.0 (1375.2, 1571.9) | 1434.6 (1218.3, 1744.5) |
| 2 | 662.6 (662.5, 662.7) | 721.6 (697.4, 747.4) | 688.4 (621.0, 772.1) |
| 3 | 341.5 (341.5, 341.6) | 355.2 (337.3, 375.0) | 345.5 (318.8, 377.0) |
| 4 | 181.9 (181.9, 181.9) | 184.3 (177.9, 191.2) | 181.7 (171.7, 192.8) |
| 5 | 92.8 (92.8, 92.8) | 92.1 (90.3, 94.1) | 89.8 (86.9, 92.9) |
| 6 | 54.1 (54.1, 54.1) | 54.7 (53.3, 56.2) | 53.5 (51.2, 55.9) |

Table S7: Results of *T_2_** fitting. *T_2_** values [µs] of 6 MnCl_2_ solutions were fitted with single exponential functions on data measured with FIDs, PETRA and HYFI. Results include the 95% confidence interval.

## Imaging parameters

|  | A | Target T2*  [us] | t_acq_  [us] | # of radial shells  in inner k-space | # of spokes in  inner k-space | # of spokes in  outer k-space | Gap  [dk] | SPI core  Radius  [dk] | N_SPI_ | TR  [ms] | NSA | Total  scan time  [s] |
| --- | --- | --- | --- | --- | --- | --- | --- | --- | --- | --- | --- | --- |
| Stack of erasers | 0 | 100 | 0 | 0 | 67056 | 51464 | 25 | 25 | 67056 | 1.00 | 1 | 119 |
|  | 0.2 | 100 | 10 | 7 | 24369 | 51462 | 25 | 5 | 552 | 1.00 | 1 | 76 |
|  | 0.6 | 100 | 40 | 4 | 11446 | 51454 | 25 | 2 | 34 | 1.00 | 1 | 63 |
|  | 1 | 100 | 2500 | 1 | 7854 | 51456 | 25 | 1 | 1 | 3.00 | 1 | 178 |
| Bone | 0 | 200 | 0 | 0 | 127284 | 205804 | 31 | 31 | 127284 | 1.00 | 8 | 2665 |
|  | 0.04 | 200 | 4 | 8 | 33372 | 205828 | 31 | 5 | 552 | 1.00 | 8 | 1914 |
| Head | 0 | 200 | 0 | 0 | 115738 | 97346 | 30 | 30 | 115738 | 0.65 | 19 | 2632 |
|  | 0.01 | 200 | 1 | 9 | 47804 | 97324 | 30 | 8 | 2294 | 0.65 | 19 | 1792 |
| Knee | x | x | x | 0 | 0 | 180960 | 1.4 | 0 | 0 | 1.00 | 1 | 181 |
|  | 0 | 500 | 0 | 0 | 530978 | 180914 | 50 | 50 | 530978 | 1.00 | 1 | 712 |
|  | 0.1 | 500 | 23 | 10 | 204632 | 180968 | 50 | 4 | 294 | 1.00 | 1 | 386 |
| Vials | 0.1 | 55 | 3 | 13 | 145350 | 31410 | 47 | 10 | 4442 | 1.00 | 1 | 177 |
|  | 0.1 | 100 | 5 | 13 | 145350 | 31410 | 47 | 10 | 4442 | 1.00 | 1 | 177 |
|  | 0.1 | 200 | 9 | 13 | 145350 | 31410 | 47 | 10 | 4442 | 1.00 | 1 | 177 |
|  | 0.1 | 400 | 18 | 13 | 145350 | 31410 | 47 | 10 | 4442 | 1.00 | 1 | 177 |
|  | 0.1 | 600 | 27 | 13 | 145350 | 31410 | 47 | 10 | 4442 | 1.00 | 1 | 177 |
|  | 0 | 55 | 0 | 0 | 441172 | 31430 | 47 | 47 | 441172 | 1.00 | 1 | 473 |
|  | 0 | 100 | 0 | 0 | 441172 | 31430 | 47 | 47 | 441172 | 1.00 | 1 | 473 |
|  | 0 | 200 | 0 | 0 | 441172 | 31410 | 47 | 47 | 441172 | 1.00 | 1 | 473 |
|  | 0 | 400 | 0 | 0 | 441172 | 31430 | 47 | 47 | 441172 | 1.00 | 1 | 473 |
|  | 0 | 600 | 0 | 0 | 441172 | 31430 | 47 | 47 | 441172 | 1.00 | 1 | 473 |

Table S8: Detailed parameter table with the amplitude coefficient *A*, the target *T_2_**, the shell acquisition time t_acq_, the number of radial shells in inner k-space, the number of spokes in inner k-space, the number of spokes in outer k-space, the gap in Nyquist dwells, the SPI core radius in Nyquist dwells, the number of excitations required in the SPI core N_SPI_, the repetition time TR, the number of sampling averages NSA and the total scan time.
